# A multicenter, randomized, open-labeled study to steer immunosuppressive and antiviral therapy by measurement of virus (CMV, ADV, HSV)-specific T cells in addition to determination of trough levels of immunosuppressants in pediatric kidney allograft recipients (IVIST01-trial): study protocol for a randomized controlled trial

**DOI:** 10.1186/1745-6215-15-324

**Published:** 2014-08-15

**Authors:** Thurid Ahlenstiel-Grunow, Armin Koch, Anika Großhennig, Cornelia Frömke, Martina Sester, Urban Sester, Christoph Schröder, Lars Pape

**Affiliations:** Department of Pediatric Kidney, Liver and Metabolic Diseases, Hannover Medical School, Carl-Neuberg-Straße 1, D-30625 Hannover, Germany; Integrated Research and Treatment Center Transplantation (IFB-Tx), Hannover Medical School, Carl-Neuberg-Straße 1, D-30625 Hannover, Germany; Department of Biostatistics, Hannover Medical School, Carl-Neuberg-Straße 1, D-30625 Hannover, Germany; Insitute for Biometry, Empidemiology and Information processing, University of Veterinary Medicine Hannover, Bünteweg 2, D-30559 Hannover, Germany; Department of Transplant and Infection Immunology, Saarland University, Kirrberger Straße Geb. 57, D_ 66421 Homburg/Saar, Germany; Department of Nephrology and Hypertension, Saarland University, Kirrberger Straße Geb. 57, D_ 66421 Homburg/Saar, Germany; Institute for Clinical Pharmacology, Hannover Medical School, Carl-Neuberg-Straße 1, D-30625 Hannover, Germany

**Keywords:** Kidney transplantation, Immunosuppression, Virus-specific T cells, Personalized immunosuppressive therapy, Viral infections, Pediatric transplantation, Cytomegalovirus, Antiviral therapy, Over-immunosuppression, Immunomonitoring, Drug-monitoring

## Abstract

**Background:**

After kidney transplantation, immunosuppressive therapy causes impaired cellular immune defense leading to an increased risk of viral complications. Trough level monitoring of immunosuppressants is insufficient to estimate the individual intensity of immunosuppression. We have already shown that virus-specific T cells (Tvis) correlate with control of virus replication as well as with the intensity of immunosuppression. The multicentre IVIST01-trial should prove that additional steering of immunosuppressive and antiviral therapy by Tvis levels leads to better graft function by avoidance of over-immunosuppression (for example, viral infections) and drug toxicity (for example, nephrotoxicity).

**Methods/design:**

The IVIST-trial starts 4 weeks after transplantation. Sixty-four pediatric kidney recipients are randomized either to a non-intervention group that is only treated conservatively or to an intervention group with additional monitoring by Tvis. The randomization is stratified by centre and cytomegalovirus (CMV) prophylaxis. In both groups the immunosuppressive medication (cyclosporine A and everolimus) is adopted in the same target range of trough levels. In the non-intervention group the immunosuppressive therapy (cyclosporine A and everolimus) is only steered by classical trough level monitoring and the antiviral therapy of a CMV infection is performed according to a standard protocol. In contrast, in the intervention group the dose of immunosuppressants is individually adopted according to Tvis levels as a direct measure of the intensity of immunosuppression in addition to classical trough level monitoring. In case of CMV infection or reactivation the antiviral management is based on the individual CMV-specific immune defense assessed by the CMV-Tvis level. Primary endpoint of the study is the glomerular filtration rate 2 years after transplantation; secondary endpoints are the number and severity of viral infections and the incidence of side effects of immunosuppressive and antiviral drugs.

**Discussion:**

This IVIST01-trial will answer the question whether the new concept of steering immunosuppressive and antiviral therapy by Tvis levels leads to better future graft function. In terms of an effect-related drug monitoring, the study design aims to realize a personalization of immunosuppressive and antiviral management after transplantation. Based on the IVIST01-trial, immunomonitoring by Tvis might be incorporated into routine care after kidney transplantation.

**Trial Registration:**

EudraCT No: 2009-012436-32, ISRCTN89806912 (17 June 2009).

## Background

After solid organ transplantation (Tx), immunosuppressive treatment disrupts the individual balance between virus replication and cellular immune response. This leads to an elevated risk of severe viral complications due to primary infection or reactivation (for example, cytomegalovirus (CMV), adenovirus (ADV), or herpes-simplex-virus (HSV)). CMV is known as a major pathogen after kidney Tx, whereas the importance of ADV and HSV is still unclear, especially in children. Because of high costs and severe side effects, antiviral treatment should be restricted to patients with high risk of viral diseases [1]. Due to missing appropriate diagnostic methods, the optimal timing of antiviral therapy/prophylaxis remains a subject for debate. Virus load and virus serology are insufficient to predict the individual risk and to decide upon the necessity and duration of antiviral prophylaxis and therapy. Virus-specific T cells (Tvis) have been shown to play a significant role in the control of virus replication. Therefore, they may serve as a prognostic marker for virus-induced diseases after Tx. Preliminary studies have found that the risk of post-transplant CMV-induced disease correlated with the individual number of CMV-Tvis. Reduced frequencies of CMV-Tvis in transplant recipients are associated with increased incidence of infectious complications [2–5]. After adult kidney Tx, symptomatic CMV reactivations are preceded by a decrease in CMV-CD4+ Tvis frequencies and an increase in CMV load [2]. Gamadia and colleagues determined the kinetics and characteristics of CMV-Tvis in the course of primary CMV infections in adult renal transplant recipients: in asymptomatic individuals the CMV-CD4+ Tvis response preceded the CMV-CD8+ Tvis response, whereas in symptomatic individuals the CMV-specific effector memory CD4+ T cell response was delayed and only detectable after antiviral therapy [6, 7]. Therefore, Tvis may represent a diagnostic tool to predict the individual risk for virus complications after solid organ Tx and enable a selective post-transplant prophylaxis. It has been shown that after Tx – the time of maximal immunosuppressive therapy – levels of Tvis are lower than later after Tx. Accordingly it may be speculated that the amount of Tvis correlates with the extent of immunosuppression. Consequently, immunosuppressive therapy might better be steered by addition of this parameter than by measuring blood levels of immunosuppressants alone.

Studies concerning CMV-Tvis after kidney Tx in adult renal transplant recipients and healthy individuals were carried out in Homburg (Saar), Germany [2, 8, 9], in Los Angeles, USA [4], in Uppsala, Sweden [10] as well as in Amsterdam, The Netherlands [6, 7, 11, 12] by cytokine flowcytometry and tetramer staining. Additionally, Sester and colleagues compared levels of CMV-Tvis in long-term kidney, heart and lung transplant recipients [3]. CMV-Tvis in adult heart recipients were also evaluated in Stanford, California, USA [5] and in Berlin, Germany [13]. In Duarte, California, USA, La Rosa and colleagues investigated CMV-Tvis response after adult liver Tx [14].

Concerning ADV-Tvis after solid organ Tx, there are only a few studies available: in Homburg (Saar), Germany, the level of ADV-Tvis were investigated in adult renal transplant recipients and healthy individuals by cytokine flowcytometry [15]. In Philadelphia, Pennsylvania, USA, Olive and colleagues analysed ADV-Tvis response of healthy adults by ELISPOT and flow cytometry [16].

No studies for HSV-Yvis after solid organ Tx have been conducted to the best of our knowledge.

The only large trial of Tvis in pediatric kidney recipients has been carried out to evaluate polyoma BK virus-specific T cells in a cooperation of Italian centers for Pediatric Nephrology and the department of virology in Basel, Switzerland [17]. No pediatric studies exist for CMV-, ADV- or HSV-Tvis after solid organ Tx. Until now, to our knowledge, no trials have been conducted that base the adoption of immunosuppressive therapy not only on drug trough levels but also on the grade of suppression of Tvis.

After solid organ Tx, immunosuppressive treatment causes an increased risk of severe viral complications. Monitoring of the grade of immunosuppression is most often performed by blood levels of immunosuppressants. Virus load and serology are insufficient to predict the individual risk of viral infections and to decide on the necessity and duration of antiviral prophylaxis and therapy. Tvis have been shown to correlate with control of virus replication as well as with the intensity of immunosuppression. Therefore, they may serve as an indicator of the level of immunosuppression as well as a prognostic marker for virus-induced diseases after Tx. Using cytokine flow cytometry we are able to monitor Tvis with specificity for different virus types.

In future this method should be established as routine use in order to develop new strategies to steer and individualize immunosuppressive therapy, to avoid severe infections, to improve post-transplant management of viral infections and to optimize the individual timing of antiviral therapy.

## Methods/design

The general aim of the trial is a prolongation of renal graft function and reduction of viral infections after kidney Tx by monitoring of Tvis followed by therapeutic intervention. Therefore, the primary endpoint is the glomerular filtration rate (GFR) (cystatin C; Schwartz and Filler formula) 2 years after Tx. Secondary endpoints are reduction in viral infections after kidney Tx, optimization of the individual timing of antiviral therapy, optimization of the immunosuppressive therapy, reduction of nephrotoxic effects of cyclosporine A (CsA) and antiviral agents by optimized dosing, and premature study discontinuations due to adverse events.

**Figure 1:**
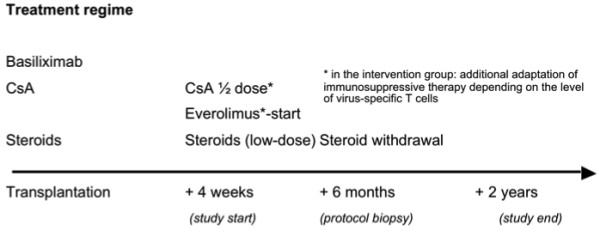
**Treatment regime.** CsA, cyclosporine A.

**Figure 2:**
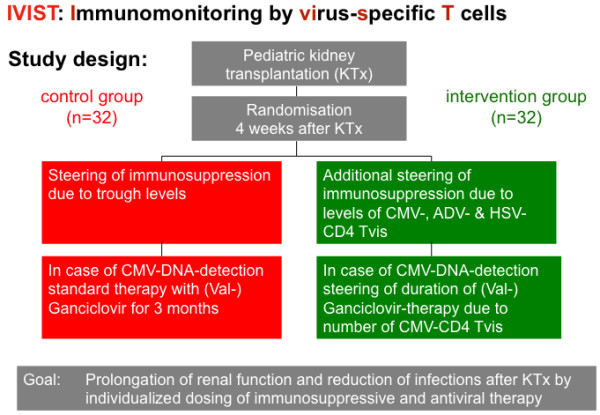
**Study design.** ADV, adenovirus; CMV, cytomegalovirus; HSV, adenovirus; Tvis, virus-specific T cells.

According to the legislation, “patients suffering from rare conditions should be entitled to the same quality of treatments as other patients” [18]. We interpret this in a way that randomized evidence for treatment decisions should be provided. Even if it is clear from the beginning that it will be difficult to achieve “significant” results at the end, the randomized comparison is obviously much less prone to bias than findings from prospective observational studies, or retrospective comparisons. In consequence the estimators and confidence intervals that will be calculated from this study will be in every case more informative than from uncontrolled studies.Therefore the study was planned as a multicenter, randomized, open-labeled study recruiting 64 children during the first 2 years after kidney Tx. This study is designed to improve the post-transplant steering of immunosuppressive drugs (CsA and everolimus) and the post-transplant management of CMV infections by quantitation of Tvis from whole blood samples. Based on specific cellular activation and induction of intracellular cytokines the levels of Tvis for different virus types (CMV, ADV, HSV) will be measured using flow cytometry (see below). In the non-intervention group, the immunosuppressive therapy is only based on classical trough level monitoring and the antiviral treatment is performed according to center practice. In the intervention group, the immunosuppressive and the antiviral therapy will be additionally adapted to the levels of Tvis. The study design is summarized in Figures 1 and 2.

Taking into account the importance of appropriate immunosuppressive medication after kidney Tx, no placebo arm will be included in this study. In both the non-intervention group and the intervention group, the immunosuppressive medication (CsA and everolimus) will be steered in the same ‘usual’ target range of trough levels due to the standard protocol. In the non-intervention group the physician arbitrarily decides on the application rate of the immunosuppressive medication within the defined normal target range of trough levels, whereas in the intervention group the immunosuppressive medication is additionally steered by measurement of Tvis levels.

### Inclusion/exclusion criteria

A total of 64 male or female children (aged 0 to 16 years) will be included. They will be enrolled if they meet the inclusion/exclusion criteria outlined in Table 1.

**Table 1:** **Selection criteria**

Inclusion criteria	
1	Patients who are males or non-pregnant females between the ages of 0 and 16 years.
2	Patients 4 weeks after kidney Tx.
3	Patients who received their first or second Tx 4 weeks ago.
4	Patients who are single-organ recipients.
5	If patients are women of childbearing potential, they must have a negative serum pregnancy test with a sensitivity equal to at least 50 mIU/ml before Tx.
6	If patients are women of childbearing potential, they must use an effective form of contraception such as the birth control pill (except mini-pill), hormonal depot injection, contraceptive hormonal patches, implanon, contraceptive hormone-containing coil or hormone-containing contraceptive vaginal ring, unless abstinence is the chosen method. In case of medical contraindication concerning the hormonal contraception, an intrauterine coil with a second contraceptive method (condom, diaphragm, spermicide) can be used. Effective contraception must be used before Tx, during therapy, and for 6 weeks following discontinuation of immunosuppressive therapy.
7	Patients’ guardians must be capable of understanding the purpose and risks of the study.
8	Patients whose guardians are willing to give written informed consent and willing to participate in and comply with the study protocol. Patients above 7 years have to agree with the study in addition to the informed consent of the legally authorized representative.
**Exclusion criteria**	
1	Patients participating in other studies or participated within the last 4 weeks before study start.
2	Patients who are highly sensitized.
3	Patients who have undergone two organ transplantations previous to the current kidney Tx (that is, two kidney transplantations, two liver transplantations, kidney and liver, or kidney and pancreas transplantation).
4	Hypersensitivity to any of the components of the medication used.
5	Patients from other centers who are not followed in the outpatient unit of the Hannover Medical School or corresponding participating centers.
6	Patients with a peak or current panel reactive antibodies >50 %.
7	Pregnant and/or lactating women and women of childbearing potential who are unwilling or unable to use contraception methods as specified.
8	Patients whose guardians do not understand the requirements of the study.
9	Patients with known positive HIV-1 or Hepatitis C virus test or the presence of Hepatitis B surface antigen.
10	Patients with malignancies or history of malignancy, despite post-transplant lymphoproliferative disease.
11	Patients who are not eligible in the opinion of the physician.
12	Significant medical history and/or treatments for cardiac, renal, neurological, hepatic, endocrine diseases, or any laboratory abnormality indicative of a significant underlying condition, that may interfere with patient’s safety, compliance, or study evaluations, according to the investigator’s opinion.

Tx, transplantation.

Patients are randomized in a 1:1 ratio to a treatment group. The randomization is stratified for CMV prophylaxis and center. Due to the open-label nature of this trial the randomization is performed centrally by the Department of Biostatistics of the Hannover Medical School to guarantee that patients are included into the trial before allocation to the treatment group is communicated. Patients are randomized in a 1:1 ratio to an intervention or a non-intervention group.

The time point of study start and randomization is 4 weeks after Tx, when the initial immunosuppressive therapy (see below) is switched to low-dose CsA and everolimus combined with low-dose steroids. The immunosuppressive therapy is steered in all patients by classical trough levels of CsA and everolimus. Patients are randomized to an intervention group with additional monitoring of Tvis or to a non-intervention group with only conservative trough level monitoring.

### Study drugs

#### Cyclosporine A

In both study groups patients are treated with CsA; according to the following schedule: the CsA start dose is 400 mg/m^2^ per day in two doses (maximal dose 250 mg); the dose will be adopted to target trough levels of 140 to 190 ng/ml (by liquid chromatography-mass spectrometry/mass-spectrometry (LC-MS/MS)) for the first 4 weeks after Tx. Afterwards, CsA dose will be reduced by 50% to a target trough level of 50 to 100 ng/ml (LC-MS/MS) (a tolerance of ±10% in the target trough level is allowed for a period of 8 weeks) when administered together with everolimus. Target CsA trough levels are reduced to 30 to 75 ng/ml (a tolerance of ±10% in the target trough level is allowed for a period of 8 weeks) 6 months after Tx.

CsA is given on a twice-daily schedule at approximately 12-hour intervals in the morning and in the evening for the complete study duration of 23 months.

Blood should be drawn within 15 minutes before the administration of the morning dose of CsA.

#### Everolimus

In both study groups patients are treated with everolimus, starting 4 weeks after Tx at 1.6 mg/m^2^ per day in two doses (maximal dose 1.5 mg). At first, target trough levels are 3 to 6 ng/ml (LC-MS/MS); 6 months after Tx the target trough levels are reduced to 2 to 5 ng/ml.

Dosing should be adjusted in all patients if the everolimus whole blood trough level is below 3 ng/ml or above 6 ng/ml (a tolerance of ±10% in the target trough level is allowed for a period of 8 weeks) and below 2 ng/ml or above 5 ng/ml (a tolerance of ±10% in the target trough level is allowed for a period of 8 weeks) 6 months after Tx.

Everolimus is administered simultaneously with CsA on a twice-daily schedule at approximately 12-hour intervals in the morning and in the evening for the complete study duration of 23 months. Blood should be drawn 15 minutes before the administration of the morning dose of everolimus.

#### Valganciclovir

In the intervention as well as in the non-intervention group, patients at high risk for primary CMV infection (CMV-seronegative recipients of CMV-seropositive organs) receive an anti-viral prophylaxis. In both groups, valganciclovir is administered prophylactically in the first 3 post-transplant months in CMV-IgG negative children who receive a kidney from a CMV-IgG positive donor.

In case of CMV infection or reactivation with relevant CMV-DNA detection, in both study groups an antiviral therapy with valganciclovir is started.

The dosing of Valganciclovir is calculated according to the following: dose (mg) = GFR × 7 × body surface area. This is applied in one single dose per day (maximum dose 1 × 900 mg). If the calculated GFR exceeds 150 ml/min per 1.73 m^2^, the dose is calculated with a GFR of 150 ml/min per 1.73 m^2^.

### Study drug adjustments

#### Immunosuppressive therapy (cyclosporine A and everolimus)

In the intervention group, the level of CD4+ Tvis (cells/μl) are detected (see below) in addition to trough level monitoring of immunosuppressants. In case of high Tvis levels (25% above the upper threshold level) the dose of immunosuppressive drugs (CsA and everolimus) will be increased 10 to 15%; in case of low Tvis levels (25% below the lower threshold level) the dose of immunosuppressive drugs (CsA and everolimus) will be decreased 10 to 15%. If the CsA or everolimus trough levels have reached the lower or upper threshold levels given above, no adaptation of CsA or everolimus doses is performed due to levels of CD4+ Tvis.

In the non-intervention group, immunosuppressive medications are only steered by classical trough levels due to the standard protocol given above.

#### Antiviral therapy (valganciclovir)

In case of a CMV infection or reactivation with relevant CMV-DNA detection, an antiviral therapy with valganciclovir is started in both study groups. In the non-intervention group valganciclovir is given for 3 months. In contrast, in the intervention group the valganciclovir therapy is carried out until a sufficient and stable number of CMV-CD4+ Tvis is found and CMV-DNA is below the detection limit.

### Concomitant immunosuppressive treatment

#### Basiliximab

The total dose of for patients weighing less than 35 kg is 20 mg intravenously, given in two doses of 10 mg each. For patients weighing 35 kg or more the recommended total dose is 40 mg intravenously, given in two doses of 20 mg each. The first 10 mg (20 mg) dose should be given within 2 hours prior to Tx surgery. The second 10 mg (20 mg) dose should be given 4 days after Tx.

#### Steroids (prednisolone)

On day 0, patients are given 300 mg/m^2^ in one dose, and then the following protocol is followed: first week after Tx, 60 mg/m^2^ in two doses/day; week 2 after Tx: 30 mg/m^2^ in two doses/day; week 3 after Tx, 15 mg/ m^2^ in one dose/day; week 4 after Tx, 12 mg/m^2^ in one dose/day; week 5 after Tx, 9 mg/m^2^ in one dose/day; week 6 after Tx, 6 mg/m^2^ in one dose/day; thereafter, final dose of 4 mg/m^2^ in one dose/day.

Patients without signs of rejection based on a protocol biopsy 6 months after kidney Tx are eligible for the controlled steroid withdrawal within 3 months. Patients with pathological changes continue steroid administration.

### Study assessments

Patients are seen and evaluated according to the flow chart given in Table 2. Patients should be encouraged to return for all evaluations as scheduled. However, a time window of 3 days for bi-weekly visits and 7 days for (bi)monthly visits is allowed.

**Table 2:** **Flow chart of study visits**

Week/month (after transplantation)	Months 1-3 (±3 days)						Months 4-12 (±7 days)			Months 13-24 (–7 days)	
	Week						Monthly (except months 6 and 12)	Months		Bimonthly (except month 24)	Month
	0	4*	6	8	10	12		6	12		24
**Visits**		**1**	**2**	**3**	**4**	**4**	**6-14**	**8**	**14**	**15-20**	**20**
Informed consent^1^		X									
Randomization		X									
Inclusion/exclusion criteria		X									
Medical history		X									
Transplantation	X										
Pregnancy test as appropriate^2^		X									
Vital signs (weight, blood pressure, pulse)		X	X	X	X	X	X	X	X	X	X
Height		X		X		X	X	X	X	X	X
Hematology (including differential count)		X	X	X	X	X	X	X	X	X	X
Chemistry panel (bilirubine., SGOT, SGPT, yGT, GlDH, CK, LDH)^3^		X					(X)		X	(X)	X
Lipid profile (cholesterol, LDL, HDL, triglycerides)		X						X	X		X
Serum creatinine, urea		X	X	X	X	X	X	X	X	X	X
Urine analysis (sticks, quantitative urine albumin and creatinine)		X	X	X	X	X	X	X	X	X	X
Trough level of CsA^4^		X	X	X	X	X	X	X	X	X	X
Trough level of everolimus^4^		X	X	X	X	X	X	X	X	X	X
CMV-, ADV-, HSV-specific T cells^5^		X	X	X	X	X	X	X	X	X	X
CMV, HSV, EBV IgG, IgM		X	X	X	X	X	X	X	X	X	X
CMV, ADV, EBV HSV-PCR		X	X	X	X	X	X	X	X	X	X
Clinical assessment		X		X	X	X	X	X	X	X	X
Protocol biopsy		X	X	X	X	X	X	X			
CsA half dose and start everolimus		X	X	X	X	X	X	X			
Start of steroid elimination		X	X	X	X	X	X	X			
Treatment of rejection^6^		X	X	X	X	X	X	X	(X)	(X)	(X)
Prior/concomitant medications		X	X	X	X	X	X	X	X	X	X
Adverse events	X	X	X	X	X	X	X	X	X	X	X

*Study start. ^1^Confirmation of informed consent prior to study start. ^2^Results must be available before study start. ^3^Chemistry panel is performed every 4 months. ^4^Levels at indicated time points will be documented on case report form (CRF) summary pages. ^5^Results at indicated time points will be documented on CRF summary page, analysis will be performed at Hannover Medical School; determination only in the intervention group. ^6^For all rejection episodes, a core renal biopsy should be performed; results of the biopsy and treatment of rejection will be documented in the medical record and recorded on the CRF. ADV, adenovirus; CK, creatine kinase; CMV, cytomegalovirus; CsA, cyclosporine A; EBV, Epstein-Barr virus; GIDH Glutamate dehydrogenase; HDL, high-density lipoprotein; HSV, herpes-simplex-virus; Ig, immunoglobulin; LDH; LDL, low-density lipoprotein; PCR, polymerase chain reaction; SGOT, serum glutamic oxaloacetic transaminase; SGPT, serum glutamic pyruvic transaminase; yGT, gamma-glutamyl-transferase.

Renal function is assessed by measuring serum creatinine, serum cystatin C, and estimated GFR by the Schwartz and Filler formula. Quantitative urine albumin and creatinine are determined as a measure of glomerular damage.

In all suspected rejection episodes, regardless of the initiation of anti-rejection treatment, a biopsy should be performed within 48 hours in the absence of medical contraindications. The histological evaluation of the biopsy will be performed locally. A biopsy-proven acute rejection is defined as a biopsy graded IA, IB, IIA, IIB, or III for the Banff 2007 classification. A protocol biopsy is performed to evaluate signs of subclinical rejection at month 6. This biopsy is only counted as protocol defined if, at this time point, no clinical signs of rejection are observed.

Chronic rejection is characterized by a slow progressive decline in renal function and is typically preceded by the histological picture of chronic allograft nephropathy. The presence of biopsy confirmed grade I, II, or III chronic allograft nephropathy by the Banff 2007 criteria is assessed on all optional biopsies obtained for clinical suspicion of chronic rejection.

Height is determined at baseline and monthly after the study start. Blood pressure will be measured at each visit and all antihypertensive medication is documented with a start and end date.

At each visit indicated in the assessment schedule, CMV-, ADV-, HSV-, and Epstein-Barr virus (EBV)-DNA as well as viral serology are determined. Analysis of the blood samples will be analyzed in each center in collaboration with the corresponding local laboratories. Active infections with CMV, EBV, ADV, HSV or other viruses are recorded with a start and end date.

In the intervention group, the number of CMV-, ADV- and HSV-Tvis are determined via cytokine flow cytometry at each visit indicated in the assessment schedule. Analysis of Tvis levels is performed at the Hannover Medical School.

### Analysis of virus-specific T cells by cytokine flow cytometry

**Figure 3:**
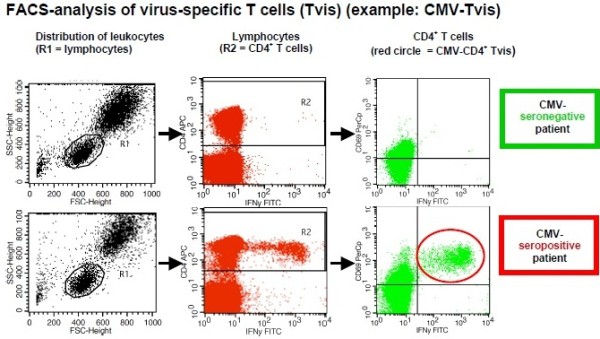
**Fluorescence-activated cell sorting analysis of virus-specific T cells (Tvis).** An example of cytomegalovirus (CMV)-Tvis is shown. FITC Fluorescein; FSC (forward scatter; IFN, interferon; SSC sideward scatter.

The method identifies CD4+ T cells in which cytokine production is stimulated by virus antigens. The analysis of Tvis is performed in four steps from heparinized whole blood:

1. Stimulation: blood leukocytes are stimulated by virus antigen. Costimulatory antibodies (CD28 and CD49d) are added to maximize the detection of T lymphocytes with a higher activation threshold. This leads to induction of intracellular cytokine production as IFNγ and TNFα. Cytokine production (IFNγ and TNFα) by Tvis is visualized by the use of Brefeldin A that inhibits granule secretion and leads to intracellular accumulation of cytokines. As a negative control, an antigen that contains no virus-specific antigen is used. The positive control is performed by *Staphylococcus aureus* enterotoxin B.
2. Fixation: erythrocytes are lysed and lymphocytes are fixed and washed.
3. Immunostaining: activated T lymphocytes are marked by fluorescent antibodies binding at CD4, CD69 and IFNγ or TNFα.
4. Fluorescence-activated cell sorting procedure: the percentage of fluorescence-marked lymphocytes is measured by flow cytometry. CD69-positive and IFNγ/TNFα-positive CD4+ T lymphocytes represent the virus-specific CD4+ T cells (CD4+ Tvis).By fluorescence-activated cell sorting analysis, the percentage of CD4+ T cells is measured that were successfully stimulated by viral antigens. The activation of T cells leads to upregulation of CD69 and intracellular production of cytokines (for example, IFNγ and TNFα) as shown in Figure  3.

### Statistical methods

The primary objective of this trial is to demonstrate a prolongation of renal graft function and (as a key secondary objective) a reduction of the number of severe viral infections/reactivation after kidney Tx by monitoring Tvis followed by therapeutic intervention. Renal function will be measured at each visit by GFR.

The primary analysis will be conducted on the intention-to-treat population. All analyses are based on two-sided hypotheses using a significance level of 5%. The precise primary hypothesis of the study is that the GFR 24 months after kidney Tx is significantly larger in the intervention group compared to the non-intervention group and will be tested by an analysis of covariance. The primary analysis model consists of the GFR 24 months after Tx as the dependent variable. Treatment strategy, including the intervention and the control treatment, a covariable including patient’s baseline GFR and two other factors (the CMV prophylaxis (yes/no) and the center) are used for stratification purposes. The estimate, as well as the two-sided 95% confidence interval, for the difference in mean intervention group/non-intervention group for the primary endpoint will be provided from the analysis of covariance model. Superiority will be concluded if the lower boundary of this confidence interval is larger than 0. Differences in the number of viral infections will be analyzed using a generalized linear model based on the negative binomial distribution. As soon as the primary hypothesis can be rejected, the reduction in the number of viral infections will be assessed as a confirmatory analysis.

Furthermore, secondary analyses will investigate the treatment effect in the strata of CMV prophylaxis and the center. In addition, interaction between treatment strategy and the CMV prophylaxis will be investigated descriptively.

### Sample size and power considerations

The clinical hypothesis is that GFR values 24 months after kidney Tx will be higher in the intervention group compared to the non-intervention group.

Due to the orphan condition under investigation, sample size calculation is feasibility driven and describes under which circumstances this trial can be formally successful.

The total sample size of this study is limited to 64 patients; that is, 32 patients per treatment group. The effect of the therapy is considered as relevant if the difference between the therapy groups in mean GFR is at least 7.5 ml/min per 1.73 m^2^ with a standard deviation of 15 ml/min per 1.73 m^2^. With these assumptions, the power to reject the null hypothesis of no difference between treatment groups with a two-sided *t*-test for two independent samples and a significance level of 5% (two-sided) is 50%. To reach a power of 80% in the same setting, a treatment effect of 10.672 ml/min per 1.73 m^2^ has to be achieved. It is assumed that stratification for CMV prophylaxis and center and adjustment for patient’s baseline will lead to an increase in power.

### Ethics statement

The study was approved by the ethics committee of Hannover Medical School (reference number 5067 M) as the leading center and the Bundesinstitut für Arzneimittel, as well as by the ethics committee of the University of Cologne, the Medical Board of Hamburg and the University of Rostsock as secondary committees who reported to the leading committee. A data safety monitoring committee (DSMC) has been implemented from the beginning of the trial consisting of a pediatric pulmonologist, a transplant surgeon and an adult nephrologist. The DSMC meets on a yearly basis and has suggested continuing with the trial until now.

Informed consent is obtained from all participating patients and their parents.

## Discussion

After kidney Tx, trough level monitoring of immunosuppressants is insufficient for individualized steering of immunosuppressive therapy and leads to over- und under-immunosuppression. In a prospective, multicenter, randomized controlled study, the IVIST01-trial will test the hypothesis that additional steering of immunosuppressive and antiviral therapy by Tvis levels will improve renal graft function and survival by individualizing post-transplant management and avoiding over-immunosuppression and drug toxicity. This novel concept of immunomonitoring by Tvis might optimize future steering of immunosuppressive and antiviral therapy and thereby become an important step towards personalized immunosuppressive and antiviral therapy in terms of an effect-related drug monitoring.

## Trial status

A total of 35 patients from two centers have been recruited (status as of 1 July 2014); 20 patients have finished the study, five patients became drop-outs (one patient in the control group died by drowning in the bath tub). All safety reports suggest continuing the trial as planned. Recruitment was slower than originally intended; therefore, the study was changed from a monocenter to a multicenter trial in 2012. Actually half of the anticipated patient numbers have been reached. The first additional center has started recruitment. With the two additional centers starting to recruit patients, the end of recruitment can be estimated for 2015.

## Abbreviations

ADV: adenovirus
CMV: cytomegalovirus
CsA: cyclosporine A
DSMC: data safety monitoring committee
EBV: Epstein-Barr virus
GFR: glomerular filtration rate
HSV: herpes-simplex-virus
IFN: interferon
Ig: immunoglobulin
LC-MS/MS: liquid chromatography-mass spectrometry/mass-spectrometry
TNF: tumor necrosis factor
Tvis: virus-specific T cells
Tx: transplantation.
